# Examination of psycho-motor development of children who were 6–36 months in the COVID-19 stay-at-home period

**DOI:** 10.1038/s41598-023-47865-4

**Published:** 2023-11-27

**Authors:** Mahmut Kılıç, Şeyda Koçak

**Affiliations:** 1 Department of Public Health, Yozgat Bozok University Faculty of Medicine, Erdoğan Akdağ Kampüsü, 66900 Yozgat, Turkey; 2 Family Health Center, Yozgat, Turkey

**Keywords:** Paediatric research, Paediatrics

## Abstract

This study aims to examine the pandemic's effect on the psycho-motor development of children aged 6–36 months during the Covid 19 pandemic period and now aged 2–5 years. This study was cross-sectional and children (n = 150) aged 2–5 years were included in the study. Data were collected using the Ankara Developmental Screening Inventory (ADSI) in 2022. The proportion of children included in the study who have general development, language-cognitive, fine motor, gross motor, and social skill-self-care development levels at a delay-suspiciously were 25.4%, 18.0%, 58.7%, 22.0%, and 25.3%, respectively. Children's overall development and specific areas of development are more positively affected by the younger age of the child. Additionally, shorter pregnancies, earlier pregnancies, and father involvement in childcare all have positive effects on child development. During the pandemic period, the fact that older children stay at home has further negatively affected their development. Fine motor development was most negatively affected.

## Introduction

On March 11, 2020, the World Health Organization (WHO) declared the Covid-19 outbreak a pandemic and a significant number of people worldwide became infected in a short period of time. Throughout this process, the Covid-19 pandemic, which affected all segments of society, deeply influenced various aspects of people's lives such as mental health, physical well-being, employment, and social interactions^1^. In situations like this, children are more affected compared to adults (traumatic events like natural disasters and pandemics). Due to the insufficient development of appropriate emotional reactions and coping mechanisms, children are more prone to experiencing stress and trauma in extraordinary circumstances compared to adults^2^.

With the global spread of the Covid-19 pandemic, many countries, including Turkey, have taken measures such as isolation and maintaining social distancing to prevent its rapid spread. These measures have resulted in significant disruptions to the normal flow of social life, particularly impacting early childhood children to some extent^3^. During the pandemic, all institutions providing early childhood care services were closed, transitioning to remote (online) education. As a result, early childhood education programs could not be implemented, depriving children of social interaction opportunities^4^. Especially preschool children, whose developmental areas are not fully matured, have difficulty understanding the restrictions during the Covid-19 period^5^. Children were forced to spend long hours in front of screens during the outbreak, negatively affecting their memory and language development. Additionally, the measures taken during the pandemic have led to a lack of physical activity in children^3^.

Play is crucial during the preschool period. Children spend their time outside of sleep hours running, jumping, and exploring their surroundings. Through play, children attempt to make sense of things around them that they don't understand. The games they play have a positive impact on their cognitive, social, emotional, language, and psychomotor development^5^. However, the resulting inadequate physical activity after these measures negatively affects children's cognitive, physical (motor), and emotional functions^3^.

In children during the Covid-19 pandemic, issues such as sleeping later at night, decreased sleep quality, irregular nutrition leading to excessive weight gain, and insufficient physical activity have been observed. Furthermore, there have been changes in spatial limitations and family activities during the pandemic period. Due to changes in routine life, emotional, social, behavioral, and language-cognitive development, as well as fine and gross motor skills in children, can undergo various alterations. In the social isolation periods, emotional changes such as anger, rebellion, helplessness, unhappiness, sadness, withdrawal, and fear of loss; behavioral disorders like excessive restlessness, aggression, finger-sucking, and stubbornness; language development problems such as difficulty speaking and expressing oneself loudly can occur^6,7^.

In a study conducted in the United States (US), it was found that children born during the pandemic scored significantly lower in verbal, motor, and general cognitive ability measurements. A cohort study conducted since 2011 indicated that those born after the pandemic began had an average IQ score 22 points lower than previous cohorts, with an average of 78 points^8^.

Gaining a comprehensive understanding of how the Covid-19 pandemic impacts children is crucial for devising effective interventions and safeguarding their well-being. Therefore, this study aims to investigate the effects of the pandemic on the psycho-motor development of children between 6 and 36 months during the pandemic, with a focus on those between the ages of 2 to 5 years during the research process.

## Materials and methods

### Study type

This research is a cross-sectional study with an analytical nature.

### Participants

The study population consists of children aged 6 to 36 months during the Covid-19 pandemic period and children aged 2 to 5 years during the research period. The sample of the study includes children who were brought by their parents or caregivers for child monitoring or examination to the Yozgat Central Family Health Center No. 5. The sample size was calculated using the GPower 3.1 program. The frequency of developmental deficits observed in children of a similar age group to the children to be studied has been used as the basis for calculating the sample size. The study relied on Büyüktaşkapu's work on children aged 1–3 years, which found that 52–55% of children had developmental deficiencies^9^. With a frequency of developmental deficiency at 0.5 (50%), effect size of 0.15, type 1 error ɑ = 0.05, and power level (1−β) of 0.80, the minimum sample size was calculated to be n = 69. The study involved 150 children and their parents or caregivers.

### Measures

Data was collected between January and June 2022 using a data collection questionnaire prepared by the researcher and the Ankara Developmental Screening Inventory (AGTE) test administered to the children. The AGTE is an assessment tool that provides systematic information about infant and child development. It is suitable for infants aged 1–3 months and children aged 12–60 months. The AGTE was developed by Erol, Sezgin, and Savaşır in 1994^10^, and its 4th revised edition was published in 2006^11^. It is tailored to our culture, designed for easy application to a large number of people in a short time. This inventory evaluates the development and skills of infants and children aged 0–6 years based on information provided by the parents, who are the people who know the child best. The information provided by parents is based on long-term observation. Thus, temporary conditions during the test administration such as illness, sleep, and fatigue, which could negatively affect the results, do not affect the family's assessment. As a result of the AGTE assessment tool and Denver Developmental Screening Inventory to three different groups, the existence of very high correlations between the agreement percentages of both scales in distinguishing normal, premature and mentally disabled groups (0.92, 0.96, 0.90, respectively) shows that the criterion-related validity of the inventory is high^10^.

The inventory allows for the early detection of infants and children at risk for developmental delay and irregularities, enabling the implementation of necessary precautions. The inventory consists of 154 questions prepared for various age groups and answered by parents as 'Yes', 'No', or 'I Don't Know'. The questions are designed to represent fine motor, gross motor, language-cognitive, and social skill-self-care development. Development scores below 30.0% of the child's score are categorized as 'Regression', scores between 20.0 and 30.0% are 'Suspect Development', scores between 20.0% and the child's chronological age are 'Normal Development', and scores above the child's chronological age line are classified as 'Advanced Development'^10^.

### Procedures

To administer the AGTE scale, the researcher received training on how to apply the scale from a child development expert and child psychologist. Before starting the study, all participants were informed about the purpose of the study and the confidentiality of the data obtained, emphasizing that the data would only be used for scientific purposes. A brief explanation of the study was provided, and those who agreed to participate were informed that the survey and test would be completed in 20–30 min.

### Statistical analysis

The data was analyzed using SPSS 25. The normal distributions of the data were visually determined by Q–Q Plots and histograms. Since the data were not normally distributed, the non-parametric tests were used to compare arithmetic means. The chi-square test was used for percentages, the Mann–Whitney test for arithmetic means. The dependent variables included AGTE scale scores and child developmental levels based on the scale. Independent variables included socio-demographic characteristics of the child and the family. Sociodemographic variables identified as significant by the AGTE scale were analyzed using multinomial logistic regression (MLR), a non-parametric technique, with a backward elimination method. In the MLR analysis, categorical variables such as gender, attendance at daycare, attending daycare during the pandemic, type of indoor activities during the pandemic, child's play area during the pandemic, mode of birth, breastfeeding education, maternal employment status, father's occupation, and father's involvement in childcare were converted into dummy variables and included in the model. The significant variables in the analysis are shown in the tables. A p-value of < 0.05 was considered statistically significant.

### Ethics approval and consent to participate

Institutional permission for the research was obtained from the Yozgat Provincial Health Directorate, and ethical approval was granted by the Yozgat Bozok University Ethics Committee (decision dated 16.02.2022, no. 30/11). Mothers were informed about the study and their informed consent was obtained. The research was carried out in accordance with the rules and ethical codes specified in the Declaration of Helsinki.

## Results

Upon analyzing the various characteristics of the children included in the study, the following findings were observed: 58.0% of the children were female, 41.3% were aged 37–48 months (with a median age of 48 months), 44.7% experienced their mothers' first pregnancy, 58.7% had a gestational period ranging from 37 to 39 weeks (with a median of 39 weeks), 39.3% had a birth weight between 3000 and 3499 g (with an average of 3105 g), 54.0% were delivered through cesarean section 85.3% were planned pregnancies. 43.6% were breastfed for duration of 2 years or longer (with a median duration of 20 months), 52.7% began walking between 12 and 14 months (with a median of 12 months), 58.0% demonstrated a better developmental level compared to their peers, as perceived by their mothers (Table 1).

**Table 1:** Children with “Delay-Suspicious” development level according to various characteristics of them.

Characteristics		Count	Col.%	GD	LCD	FMD	GMD	SSD
				n = 38%	n = 27%	n = 88%	n = 33%	n = 38%
Sex	Girls	87	58.0	23.0	19.5	55.2	21.8	14.9
	Boys	63	42.0	28.6	15.9	63.5	22.2	39.7
Children age groups (month)	28–36	41	27.3	17.1	9.8	41.5	7.3	26.8
	37–48	62	41.3	27.4	16.1	66.1	30.6	22.6
	≥ 49	47	31.3	29.8	27.7	63.8	23.4	27.7
Interval between pregnancies (year)	First pregnancy	67	44.7	19.4	13.4	56.7	17.9	23.9
	≤ 2	21	14.0	28.6	23.8	42.9	19.0	9.5
	3–5	27	18.0	14.8	7.4	44.4	18.5	14.8
	> 5	35	23.3	42.9	31.4	82.9	34.3	45.7
Gestational age (weeks)	< 37	14	9.3	14.3	7.1	50.0	21.4	35.7
	37–39	88	58.7	27.3	18.2	60.2	25.0	22.7
	≥ 40	48	32.0	25.0	20.8	58.3	16.7	27.1
Birth weight (gram)	< 2500	17	11.3	29.4	11.8	58.8	35.3	29.4
	2500–2999	34	22.7	26.5	26.5	64.7	26.5	26.5
	3000–3499	59	39.3	25.4	11.9	54.2	20.3	25.4
	≥ 3500	40	26.7	22.5	22.5	60.0	15.0	22.5
Type of birth	Normal	69	46.0	33.3	24.6	62.3	26.1	21.7
	Cesarean section	81	54.0	18.5	12.3	55.6	18.5	28.4
Wanting to be pregnant	Desired pregnancy	128	85.3	23.4	18.8	57.8	21.1	25.0
	Unwanted pregnancy	22	14.7	36.4	13.6	63.6	27.3	27.3
Breastfeeding duration (months)	< 6	30	20.1	26.7	23.3	46.7	16.7	23.3
	6–11	22	14.8	45.5	27.3	68.2	27.3	36.4
	12–23	32	21.5	18.8	12.5	68.8	28.1	25.0
	≥ 24	65	43.6	21.5	15.4	56.9	20.0	23.1
Time to start walking (months)	< 12	47	31.3	25.5	14.9	55.3	21.3	17.0
	12–14	79	52.7	21.5	16.5	58.2	17.7	29.1
	≥ 15	24	16.0	37.5	29.2	66.7	37.5	29.2
Child's developmental level by family	Better than its peers	87	58.0	19.5	13.8	57.5	21.8	19.5
	Same as their peers	54	36.0	29.6	20.4	63.0	24.1	31.5
	Backward than their peers	9	6.0	55.6	44.4	44.4	11.1	44.4
	Total	150	100.0	25.3	18.0	58.7	22.0	25.3
Type of activity at home in the pandemic	Gaming with siblings	23	15.3	30.4	17.4	65.2	30.4	21.7
	Indoor activities	102	68.0	23.5	14.7	54.9	18.6	29.4
	Other	25	16.7	28.0	32.0	68.0	28.0	12.0
Screen time in pandemic (hours)	≤ 2	73	48.7	20.5	15.1	52.1	16.4	19.2
	3–4	50	33.3	28.0	20.0	62.0	24.0	40.0
	≥ 5	27	18.0	33.3	22.2	70.4	33.3	14.8
Going to daycare in the pandemic	Not gone	140	93.3	25.7	17.9	59.3	22.9	25.7
	Gone	10	6.7	20.0	20.0	50.0	10.0	20.0
Currently going to daycare	No	120	80.0	25.8	18.3	59.2	22.5	24.2
	Yes	30	20.0	23.3	16.7	56.7	20.0	30.0
Playing at home in the pandemic	No	14	9.3	21.4	21.4	57.1	14.3	14.3
	Yes	136	90.7	25.7	17.6	58.8	22.8	26.5
Playing around the building in the pandemic	No	135	90.0	25.2	17.0	59.3	22.2	27.4
	Yes	15	10.0	26.7	26.7	53.3	20.0	6.7
Playing games in the village/garden in the pandemic	No	131	87.3	25.2	16.8	60.3	22.1	26.7
	Yes	19	12.7	26.3	26.3	47.4	21.1	15.8
	Total	150	100.0	25.3	18.0	58.7	22.0	25.3

GD: general development; LCD: language-cognitive development; FMD: fine motor development; GMD: gross motor development; SSD: social skills-self-care development.

During the period of restricted outdoor activities for children due to the pandemic: 68.0% of families engaged in indoor activities with their children, 51.3% reported that their children spent 3 h or more per day in front of screens such as TVs, tablets, computers, and cell phones, 93.3% of the children did not attend daycare, 90.7% played at home, 10.0% played around the building, and 12.7% played in the village or garden (Table 1).

Regarding fathers' involvement in childcare among the children in the study: 68.0% of fathers participated in child feeding, 67.3% participated in dressing the child, 64.0% participated in bathing the child, 72.0% participated in playing with the child, 66.0% assisted in putting the child to sleep (Table 2).

**Table 2:** Children with "Delay-Suspicious" development level according to various characteristics of their families.

		Sayı	%	GD	LCD	FMD	GMD	SSD
				n = 38%	n = 27%	n = 88%	n = 33%	n = 38%
Mother’s age (years)	21–29	47	31.3	29.8	21.3	57.4	17.0	23.4
	30–34	52	34.7	21.2	15.4	55.8	25.0	21.2
	35–39	39	26.0	28.2	17.9	61.5	25.6	30.8
	≥ 40	12	8.0	16.7	16.7	66.7	16.7	33.3
Father’s age (years)	25–29	14	9.3	28.6	14.3	50.0	14.3	35.7
	30–34	61	40.7	18.0	14.8	57.4	21.3	16.4
	35–39	33	22.0	33.3	21.2	51.5	21.2	21.2
	≥ 40	42	28.0	28.6	21.4	69.0	26.2	38.1
Mother’s education levels	Primary school	26	17.3	30.8	11.5	73.1	30.8	23.1
	Middle school	24	16.0	45.8	37.5	70.8	25.0	25.0
	High school	51	34.0	29.4	25.5	54.9	21.6	27.5
	University	49	32.7	8.2	4.1	49.0	16.3	24.5
Father’s education levels	Primary school	28	18.7	35.7	32.1	60.7	28.6	10.7
	Middle school	24	16.0	20.8	16.7	62.5	12.5	20.8
	High school	40	26.7	37.5	25.0	70.0	30.0	37.5
	University	58	38.7	13.8	6.9	48.3	17.2	25.9
Mother's working status	Not working	120	80.0	28.3	19.2	61.7	23.3	24.2
	Working	30	20.0	13.3	13.3	46.7	16.7	30.0
Father occupation	Not working- unemployed	22	14.7	22.7	18.2	63.6	13.6	18.2
	Public employee	46	30.7	17.4	8.7	50.0	23.9	30.4
	Tradesman-farmer	23	15.3	21.7	26.1	52.2	26.1	4.3
	Worker in private	59	39.3	33.9	22.0	66.1	22.0	32.2
Number of children in the family	1	47	31.3	20.5	10.3	61.5	20.5	20.5
	2	79	52.7	23.5	17.6	50.0	20.6	26.5
	≥ 3	24	16.0	32.6	25.6	69.8	25.6	27.9
Father helping child feeding	None	48	32.0	29.2	18.8	58.3	27.1	27.1
	Yes	102	68.0	23.5	17.6	58.8	19.6	24.5
Father helping child dress up	None	49	32.7	30.6	20.4	59.2	26.5	30.6
	Yes	101	67.3	22.8	16.8	58.4	19.8	22.8
Father helping child bath	None	54	36.0	29.6	20.4	59.3	25.9	27.8
	Yes	96	64.0	22.9	16.7	58.3	19.8	24.0
Father playing with the child	None	42	28.0	31.0	21.4	66.7	23.8	31.0
	Yes	108	72.0	23.1	16.7	55.6	21.3	23.1
Father helping child sleep	None	51	34.0	31.4	19.6	62.7	27.5	29.4
	Yes	99	66.0	22.2	17.2	56.6	19.2	23.2
Income levels (TL)	< 4000	24	16.0	45.8	29.2	79.2	20.8	20.8
	4000–4999	45	30.0	33.3	20.0	68.9	24.4	35.6
	5000–6999	25	16.7	24.0	20.0	56.0	28.0	20.0
	7000–9999	23	15.3	13.0	13.0	39.1	30.4	13.0
	≥ 10,000	33	22.0	9.1	9.1	45.5	9.1	27.3
	Total	150	100.0	25.3	18.0	58.7	22.0	25.3

GD: general development; LCD: language-cognitive development; FMD: fine motor development; GMD: gross motor development; SSD: social skills-self-care development.

When examining the general developmental levels of the children, it was observed that 25.4% were at the delay-suspect level, 48.0% were at the normal level, and 26.7% were at the advanced level (Table 3). An multinomial logistic regression analysis indicated that for overall developmental levels, being at the normal level was influenced by increased family income and decreased parental age (borderline significance at p = 0.052). Advanced developmental levels were influenced by decreased child age, increased family income, and father's involvement in the child's play activities. These variables accounted for 26.9% of the variance in overall developmental level. Other variables included in the analysis were not found to be statistically significant (Table 4).

**Table 3:** Developmental levels of children by sex.

		Sex						X^2^
		Girl		Boy		Total		
		Count	Col.%	Count	Col.%	Count	Col.%	p
General Development	Delay	2	2.3	5	7.9	7	4.7	2.779
	Suspicious	18	20.7	13	20.6	31	20.7	0.427
	Normal	44	50.6	28	44.4	72	48.0	
	Advanced	23	26.4	17	27.0	40	26.7	
	Mean ± SD^a^	131.9	8.0	129.6	10.0	130.9	8.9	P = 0.184
Language-cognitive development	Delay	4	4.6	4	6.3	8	5.3	1.464
	Suspicious	13	14.9	6	9.5	19	12.7	0.691
	Normal	42	48.3	29	46.0	71	47.3	
	Advanced	28	32.2	24	38.1	52	34.7	
	Mean ± SD^a^	51.5	4.6	51.2	5.8	51.4	5.1	P = 0.933
Fine motor development	Delay	23	26.4	23	36.5	46	30.7	1.892
	Suspicious	25	28.7	17	27.0	42	28.0	0.595
	Normal	28	32.2	17	27.0	45	30.0	
	Advanced	11	12.6	6	9.5	17	11.3	
	Mean ± SD^a^	20.7	2.0	20.1	2.2	20.5	2.1	**P = 0.036**
Gross motor development	Delay	9	10.3	6	9.5	15	10.0	0.074
	Suspicious	10	11.5	8	12.7	18	12.0	0.995
	Normal	44	50.6	32	50.8	76	50.7	
	Advanced	24	27.6	17	27.0	41	27.3	
	Mean ± SD^a^	23.6	.5	23.5	0.6	23.6	0.5	P = 0.052
Social skill and self-care developmental	Delay	6	6.9	6	9.5	12	8.0	13.749
	Suspicious	7	8.0	19	30.2	26	17.3	**0.003**
	Normal	48	55.2	26	41.3	74	49.3	
	Advanced	26	29.9	12	19.0	38	25.3	
	Mean ± SD^a^	36.0	2.7	35.0	2.5	35.6	2.7	**P = 0.006**
	Total	87	100.0	63	100.0	150	100.0	

^a^Mann–Whitney test.

p < 0.05 values are in bold.

**Table 4:** Estimation of parameters affecting children's development level by MLR (backward elimination) analysis.

		B	Std. error	P	O.R.	95% confidence interval for O.R.	
						Lower	Upper
General^a^ Reference category: Delay-Suspect							
Normal	Intercept	− 0.837	1.564	0.593			
	Age	− 0.046	0.024	**0.052**	0.955	0.912	1.000
	Family income levels	0.531	0.173	**0.002**	1.700	1.211	2.388
	Father playing with the child = none	0.208	0.467	0.655	1.232	0.493	3.075
Advanced	Intercept	1.128	1.756	0.521			
	Age	− 0.092	0.029	**0.002**	0.912	0.861	0.966
	Family income levels	0.805	0.206	**0.000**	2.237	1.494	3.349
	Father playing with the child = none	− 1.502	0.697	**0.031**	0.223	0.057	0.874
Language-cognitive^b^ Reference category: Delay-Suspect							
Normal	Intercept	5.307	2.044	0.009			
	Age	− 0.077	0.030	**0.010**	0.926	0.873	0.982
	Mother’s education level	0.227	0.243	0.350	1.254	0.780	2.018
	Interpregnancy Interval	− 0.125	0.078	0.109	0.883	0.758	1.028
	Breastfeeding duration	0.027	0.025	0.273	1.028	0.979	1.079
	Time to start walking	− 0.127	0.071	0.073	0.881	0.767	1.012
	Breastfeeding education = none	0.122	0.511	0.812	1.129	0.415	3.076
Advanced	Intercept	6.128	2.429	0.012			
	Age	− 0.144	0.035	**0.000**	0.866	0.809	0.928
	Mother’s education level	0.721	0.285	**0.011**	2.057	1.177	3.595
	Interpregnancy Interval	− 0.192	0.089	**0.031**	0.825	0.692	0.983
	Breastfeeding duration	0.084	0.030	**0.006**	1.087	1.024	1.154
	Time to start walking	− 0.242	0.096	**0.011**	0.785	0.651	0.947
	Breastfeeding education = none	1.342	0.612	**0.028**	3.828	1.153	12.706
Fine motor^c^ Reference category: Delay-Suspect							
Normal	Intercept	4.232	2.112	0.045			
	Age	− 0.069	0.023	**0.003**	0.933	0.891	0.977
	Mother’s age	0.059	0.071	0.403	1.061	0.924	1.218
	Father’s age	− 0.111	0.073	0.129	0.895	0.776	1.033
	Time to start walking	− 0.123	0.077	0.113	0.885	0.760	1.029
	Family income levels	0.330	0.167	**0.049**	1.391	1.002	1.931
	Father helping child feeding = none	2.058	0.843	**0.015**	7.827	1.500	40.837
	Father playing with the child = none	− 2.218	0.863	**0.010**	0.109	0.020	0.591
Advanced	Intercept	− 2.543	3.012	0.399			
	Age	− 0.087	0.035	**0.012**	0.916	0.856	0.981
	Mother’s age	− 0.215	0.113	**0.058**	0.807	0.646	1.007
	Father’s age	0.200	0.102	**0.050**	1.222	1.000	1.492
	Time to start walking	− 0.253	0.144	0.079	0.776	0.585	1.030
	Family income levels	1.388	0.345	**0.000**	4.007	2.040	7.874
	Father helping child feeding = none	− 0.206	1.324	0.876	0.813	0.061	10.900
	Father playing with the child = none	0.752	1.410	0.594	2.121	0.134	33.619
Gross motor Reference category: Delay-Suspect							
Normal	Intercept	− 0.672	1.770	0.704			
	Age	− 0.016	0.026	0.532	0.984	0.934	1.036
	Number of children in the family	− 0.086	0.277	0.755	0.917	0.533	1.577
	Interpregnancy interval	− 0.103	0.076	0.175	0.903	0.778	1.047
	Birth weight	0.001	0.000	**0.030**	1.001	1.000	1.002
	Father occupation = not working- unemployed	0.909	0.767	0.236	2.481	0.552	11.156
	Father occupation = public employee	− 0.289	0.500	0.564	0.749	0.281	1.998
Advanced	Intercept	1.585	2.087	0.448			
	Age	− 0.148	0.034	**0.000**	0.862	0.807	0.921
	Number of children in the family	0.659	0.320	**0.040**	1.934	1.032	3.624
	Interpregnancy Interval	− 0.204	0.096	**0.033**	0.815	0.676	0.984
	Birth weight	0.001	0.001	**0.026**	1.001	1.000	1.002
	Father occupation = not working-unemployed	2.073	0.899	**0.021**	7.945	1.363	46.313
	Father occupation = public employee	1.085	0.629	0.084	2.961	0.863	10.155
Social skill and self-care Reference category: Delay-Suspect							
Normal	Intercept	3.902	1.867	0.037			
	Age	− 0.028	0.025	0.269	0.972	0.925	1.022
	Interpregnancy interval	− 0.162	0.070	**0.021**	0.850	0.741	0.976
	Time to start walking	− 0.095	0.073	0.192	0.909	0.788	1.049
	Sex = girl	1.368	0.462	**0.003**	3.927	1.588	9.708
	Father helping child feeding = none	1.766	1.261	0.161	5.850	0.494	69.288
	Father helping child dress up = none	− 2.161	1.245	0.083	0.115	0.010	1.322
	Father occupation = tradesman-farmer	2.553	1.444	0.077	12.846	0.759	217.533
	Type of activity at home in the pandemic = indoor activities	− 1.231	0.556	**0.027**	0.292	0.098	0.869
Advanced	Intercept	6.996	2.260	0.002			
	Age	− 0.083	0.030	**0.006**	0.921	0.867	0.977
	Interpregnancy interval	− 0.159	0.082	**0.054**	0.853	0.726	1.003
	Time to start walking	− 0.239	0.103	**0.020**	0.788	0.644	0.963
	Sex = girl	1.611	0.569	**0.005**	5.007	1.641	15.274
	Father helping child feeding = none	2.989	1.521	**0.049**	19.867	1.008	391.620
	Father helping child dress up = none	− 3.146	1.519	**0.038**	0.043	0.002	0.844
	Father occupation = tradesman-farmer	3.758	1.483	**0.011**	42.882	2.344	784.667
	Type of activity at home in the pandemic = indoor activities	− 1.367	0.651	**0.036**	0.255	0.071	0.914

Gross motor: Goodness-of-Fit p = 0.278, Nagelkerke R^2^ = 0.327.

Social skill and self-care: Goodness-of-Fit p = 0.486, Nagelkerke R^2^ = 0.330.

Independent variables: Continuous or ordinal variables: Child's age, gestational interval, gestational age, birth weight, maternal age, father's age, family total income level, father's education level, mother's education level, walking time. Dummy variables: Gender, going to daycare, going to daycare during the pandemic, the type of spending time at home during the pandemic, the child's playground during the pandemic, the mode of delivery, breastfeeding education, mother's employment status, father's occupation, father's participation in childcare.

^a^Goodness-of-Fit p = 0.497, Nagelkerke R^2^ = 0.269. ^b^Goodness-of-Fit p = 0.077, Nagelkerke R^2^ = 0.336, ^c^Goodness-of-Fit p = 0.909, Nagelkerke R^2^ = 0.365.

p < 0.05 values are in bold.

Similarly, concerning language-cognitive development, 18.0% were at the delay-suspect level, 47.3% were at the normal level, and 34.7% were at the advanced level (Table 3). The multinomial logistic regression analysis revealed that normal language-cognitive development was influenced solely by the child's age, while advanced development was influenced by the child's age, increased maternal education, shorter pregnancy intervals or first pregnancies, longer breastfeeding durations, earlier walking ages, and lack of breastfeeding education. These variables accounted for 33.6% of the variance in language-cognitive development. Other variables included in the analysis were not found to be statistically significant (Table 4).

Concerning fine motor development, 58.7% were at the delay-suspect level, 30.0% were at the normal level, and 11.3% were at the advanced level (Table 3). The multinomial logistic regression analysis indicated that normal fine motor development was influenced by the child's age, increased family income, and father's lack of participation in feeding and engagement in play activities. Advanced fine motor development was influenced by the child's age, decreased maternal age, increased paternal age, increased family income (p < 0.05), and an earlier walking age (borderline significance at p = 0.079). These variables accounted for 36.5% of the variance in fine motor development. Other variables included in the analysis were not statistically significant (Table 4).

In terms of gross motor development, 22.0% were at the delay-suspect level, 50.7% were at the normal level, and 27.3% were at the advanced level (Table 3). The multinomial logistic regression analysis revealed that normal gross motor development was influenced solely by increased birth weight, while advanced development was influenced by increased birth weight, decreased child age, increased number of siblings, shorter pregnancy intervals or first pregnancies, and father's unemployment (p < 0.05). These variables accounted for 32.7% of the variance in gross motor development (Table 4).

Lastly, when examining social skill and self-care developmental levels, 25.3% were at the delay-suspect level, 49.3% were at the normal level, and 25.3% were at the advanced level (Table 3). The multinomial logistic regression analysis indicated that for normal social skill and self-care development, being at the normal level was influenced by shorter pregnancy intervals or first pregnancies, being female, and engaging in indoor activities for children during the pandemic (p < 0.05). Advanced social skill development was influenced by the child's age, shorter pregnancy intervals or first pregnancies (borderline significance at p = 0.054), earlier walking age, being female, father's lack of participation in feeding, father's assistance in dressing, father's profession as a tradesperson-farmer, and engaging in indoor activities for children during the pandemic (p < 0.05). These variables accounted for 33.0% of the variance in social skill development (Table 4).

## Discussion

This study examined how the psycho-motor development of children aged 2 to 5 years, specifically those within the 6–36 months age range, was impacted during the Covid-19 quarantine period. A review of the literature revealed a limited number of studies investigating the effects of the pandemic on the psycho-motor development of preschool children.

The Covid-19 pandemic brought about changes in family dynamics, affecting children's school routines, parents' work schedules, and consequently, family lifestyles. These measures significantly influence children's mental well-being and psycho-motor development^12^.

### General psychomotor development of children

Among the children included in the study, it was observed that 25.4% (4.7% delayed) were at the delay-suspect level, 48.0% were at the normal level, and 26.7% were at the advanced general developmental level. Normal developmental levels were influenced by an increase in family income and a decrease in child age, while advanced developmental levels were influenced by a decrease in child age, an increase in family income, and fathers' participation in children's play activities (Table 4). Similar to our study, Şengönül's research also concluded that an increase in family income positively affected child development^13^. As expected, research has shown that a decrease in family income negatively affects children's development^14–17^.

Examining studies conducted in Turkey, our findings align with the notion that children's overall development is positively impacted when they engage in interactive play with their fathers^18^. Türkoğlu and colleagues' qualitative study in 2013, involving interviews with 12 fathers, revealed that all fathers played with their children, and 12 of the participating fathers (100%) believed that spending quality time with their children positively affected the child's social-emotional, language, psycho-motor, cognitive, and self-care development^19^. The study concluded that playing with fathers positively influenced children's development, aligning with our study's findings^20^.

Despite the pandemic's ongoing impact, it was found that 4.7% of the children included in the study delayed developmental regression. A study conducted on infants in Vietnam (2010) identified psychomotor developmental delay in 8.3% of the infants^21^. Another study in Egypt, conducted just before the pandemic (September 2019–March 2020) among children aged 2 to 36 months, found developmental delay in 9.3% of the children^22^. Our findings appear to be lower than the results of these two studies. This variation could be attributed to differences in the level of development between countries.

### Language-cognitive development of children

Language-cognitive development is a critical aspect of child development, as it forms the foundation for communication, learning, and social interaction. By understanding the factors that influence language-cognitive development and providing children with the support they need, caregivers and educators can help them reach their full potential^7^. Among the children included in the study, it was found that 18% (5.3% delayed) exhibited delay-suspect levels of language-cognitive development, 47.3% were at the normal level, and 34.7% were at the advanced level. A decrease in child age influenced normal language-cognitive development, while a decrease in child age, an increase in maternal education, short or first pregnancies, extended breastfeeding duration, an earlier age of walking, and the absence of breastfeeding education all contributed to advanced language-cognitive development (Table 4). Our study also revealed that an increase in breastfeeding duration positively affected language-cognitive development, which is consistent with the literature. In a study by Özbilgin et al., infants exclusively breastfed had significantly higher scores in the language-cognitive development parameter (104.2) compared to formula-fed infants (85.7)^23^. Similarly, higher education levels among mothers correlated with higher language-cognitive development scores in multiple studies^24,25^.

In our study, it was observed that a decrease in child age positively influenced both normal and advanced levels of language-cognitive development. Walker found that in children aged 3–5 years, those with younger ages had lower scores for negative communication^26^. Similarly, Karoğlu and Ünüvar reported higher language-cognitive development scores in children aged 61–72 months compared to those aged 49–60 and 36–48 months^27^. Yıldırım and Koçak also noted that language-cognitive development progressed in parallel with age in their study involving 300 children. This aligns with our findings that the mean scores for language-cognitive development increase with age. In our study, children aged 28–36 months with advanced language-cognitive development accounted for 43.9%, whereas this rate was only 21.3% for those aged 49 months or older. While the positive effect of decreased age on language-cognitive development might seem contradictory, this result aligns with the appropriate developmental level based on the child's age. It is possible that older children were more negatively impacted in terms of language-cognitive development during the pandemic, as this period could be crucial for their development^24^.

Our study, like Eryılmaz et al.'s research, found that language skills in children aged 36–72 months were not affected by the number of siblings. Quality of attention and time spent are more critical within the family context than the number of siblings^28^. Furthermore, in larger families, the presence of multiple individuals for interaction and communication beyond parents can positively influence language development. A rich stimulating environment, in terms of both cultural opportunities and interaction within the child's surroundings, plays a significant role in vocabulary development. Limited vocabulary development may result from a lack of stimulating environment, restricted vocabulary within the family, absence of a proficient language model, and inadequate verbal communication with children.

Our study found a contrasting relationship between screen time during the pandemic and language-cognitive development; however, this relationship was not significant in regression analysis. Decreased screen time correlated with higher levels of language-cognitive development. This result shows parallelism with Gökçay et al.'s study^29^. They observed that children who watched television for more than two hours a day had a higher prevalence of language-cognitive delays (50%) compared to those who watched for less than two hours (31%) according to the Denver Developmental Screening Test. Similarly, our study found that children exposed to screens for 5 h or more had advanced language-cognitive development at a rate of 22.2%, while this rate was 37.0% for those who watched for less than 2 h. While our findings align with previous research, the lack of significance in the regression analysis could be attributed to the greater importance of other factors.

### Fine motor development of children

Among the children included in the study, it was found that 58.7% (30.7% delayed) exhibited delay-suspect levels of fine motor development, 30% were at the normal level, and 11.3% were at the advanced level. Normal fine motor development was influenced by decreasing child age, increasing family income, fathers not assisting in child feeding, and fathers participating in the child's games. On the other hand, advanced fine motor development was positively influenced by decreasing child age, decreasing maternal age, increasing paternal age, and increasing family income (Table 4). A study conducted in Egypt (2020) with children aged 2–36 months found that there was at least 1.0% deficiency in fine motor skills and that children with higher maternal education exhibited higher levels of fine motor development^22^. Our study's findings also support the positive impact of increasing maternal education and decreasing maternal age on fine motor development, even though the significance of maternal education was not established in the regression analysis.

During the Covid-19 pandemic, children's fine motor development was positively influenced by an increase in monthly family income (Suspect development rate: 79.2% in families with income ≤ 4000 TL and 45.5% in families with income ≥ 10,000 TL). Limited research has been conducted to investigate the causes of children's motor development delays. Avşar, İbiş, and Aktuğ found that children from lower socioeconomic backgrounds had significantly lower motor development scores, while there was no difference among children from upper and middle socioeconomic backgrounds^30^. Motor development is affected by factors such as the materials children play with and their nutrition, which aligns with our results.

Marsiglio concluded in his research that as fathers' age increased, they took on a more active role in child care and contributed more to children's development^31^. In contrast, Özcebe et al. found in a study involving 119 fathers that paternal age had no impact on child care^32^. In our study, increasing paternal age and fathers engaging in play with their children positively influenced fine motor development. Adult fathers, as they advance in their careers, may contribute more to their children's development compared to younger fathers, thus positively influencing their development. Previous studies have shown that children who engage in play and spend quality time with their fathers tend to have better development and higher IQ scores. Similarly, preschool children who receive attention from their fathers tend to exhibit greater patience and cope better with stress and difficulties^33,34^. Likewise, during the Covid-19 pandemic, children's normal fine motor development was positively influenced by playing with their fathers and spending time with them. Notably, children whose fathers did not support their feeding exhibited normal fine motor development. Allowing children to perform tasks independently during meals and tasks that require skill, such as using cutlery and glasses, may contribute to normal fine motor development.

In our study, it was found that decreasing maternal age was associated with advanced fine motor development in children. Among mothers aged 21–29, 31.9% of their children exhibited normal fine motor development, while this rate dropped to 16.7% for mothers aged 40 and above. Özyürek et al. found that decreasing maternal age was associated with increased problem-solving skills in children. Children with mothers aged 26–30 had a higher average score on the Problem Solving Skill Scale (36.21) compared to children with mothers aged 31–35 (24.79)^35^. As there is a parallel relationship between children's problem-solving skills and fine motor development, the obtained results support our study.

### Gross motor development of children

In our study, 22.0% (10% delayed) of children exhibited delay-suspect levels of gross motor development, 50.7% were at the normal level, and 27.3% were at the advanced level. Normal gross motor development was only influenced by an increase in birth weight, while advanced development was positively influenced by increased birth weight, decreasing child age, increasing number of siblings, shorter inter pregnancy interval or being the first pregnancy, and the father being unemployed (Table 4). A study conducted in Egypt (2020) with children aged 2–36 months found that 3.1% exhibited deficiencies in gross motor skills^22^. When examining studies conducted in Turkey, it is evident that the development of gross motor skills increases as children's age increases. Karoğlu and Ünüvar found in a study with 151 children that children aged 61–72 months had higher "gross motor" skills compared to children aged 49–60 and 36–48 months^27^. These studies only collected scale scores, and developmental levels were not classified based on age. In our study, it was found that during the pandemic lockdown period, decreasing child age was associated with advanced gross motor development. Among children aged 28–36 months, 46.3% exhibited advanced gross motor development, while only 8.5% of children aged 49 months and above showed advanced development. According to our research findings, the restrictions of lockdown and quarantine during the pandemic, especially for children aged 30–60 months, hindered activities that support gross motor development, such as running, kicking a ball, catching a ball, jumping on one foot, changing speed while running, climbing up and down stairs quickly, aiming and throwing objects, and riding a four-wheeled bicycle, due to being confined to the home environment. Literature review indicates limited research on factors influencing children's gross motor development during the pandemic, making these findings important.

Shorter inter pregnancy intervals or being the first pregnancy increase the likelihood of a child having advanced gross motor development. This may result in shorter intervals between children, reducing the time the mother spends on care and attention, and forcing children to perform motor activities that require gross motor skills independently.

When looking at studies that examine the relationship between children's birth weight and gross motor development, it is found that similar results were obtained in our study, indicating a proportional relationship between increasing birth weight and increasing gross motor development. Bağcı and Egemen^36^ conducted a study with 95 low-birth-weight (LBW) and 108 normal birth weight babies and found that LBW babies exhibited slower gross motor development compared to babies with normal birth weights. Specifically, motor skills such as sitting, crawling, and walking were acquired later in LBW babies compared to those with normal birth weights^36^. Sürmeli Döven et al. found that there was a significant deficiency in social, fine motor, gross motor, and language skills in low-birth-weight children compared to normally born healthy children^37^. Similarly, our study supports this result; while 15.0% of children born weighing 3500 g or more exhibited suspect development, 35.3% of children born weighing less than 2500 g exhibited suspect development. In the same study, gross motor development based on the Bayley III Developmental Assessment Scale was higher in LBW children than 24% of their peers, while this rate was 71.5% for healthy children born on time^37^. Similarly, in our study, 37.5% of children with birth weights above 3500 g exhibited advanced gross motor development, while 23.5% of those born below 2500 g exhibited advanced development.

### Social skills and self-care developmental of children

Upon examining the social skills and self-care developmental levels of the children included in the study, it was found that 25.3% exhibited delay-suspect levels (8.0% delayed), 49.3% were at the normal level, and 25.3% were at the advanced level (Table 3). Factors such as a shorter interval between pregnancies, being female, engaging in indoor activities with family during the pandemic, were found to influence children's social skills and self-care developmental levels to be normal (p < 0.05). On the other hand, factors such as a decrease in the child's age, a shorter interval between pregnancies (p = 0.054), an earlier age of walking, being female, the father not assisting in feeding, the father assisting in dressing, the father's occupation as a tradesperson or farmer, engaging in indoor activities for children during the pandemic were found to impact the child's social skills and self-care developmental levels to be advanced (p < 0.05) (Table 4).

Taşdemir Yiğitoğlu et al.^38^ conducted a study using the "Self-Care Skills Control List" scale on children and emphasized that female children were more advanced in meeting self-care needs such as eating and dressing compared to male children, aligning with our study. However, no significant difference was found based on gender in terms of personal care skills^38^. In another study, the scores obtained from the "Social Skills Scale Based on Gender" indicated that female children (43.65) managed their emotions better than male children (39.26), supporting the idea of gender-related differences in social skills^39^. In our study, 29.9% of female children demonstrated advanced social skills and self-care development, while 19.0% of male children exhibited advanced development. These results suggest that female children tend to show more advanced development in social skills and self-care, possibly due to gender-specific societal roles and the influence of female role models.

Regarding the presence of siblings, Ogelman and Sarıkaya applied the "Child Behavior Scale" to children aged 5–6 and found that children with siblings (16.33) scored higher in social skills compared to those without siblings (14.20)^40^. However, some studies, including Polat and Yağbasan who used the "Marmara School Readiness Scale" and Dinçer et al. who examined self-care skills, did not find significant differences in self-care skills based on the presence of siblings^41,42^. In our study, the presence of siblings did not yield statistically significant results on children's social skills and self-care developmental levels.

The COVID-19 pandemic led to children spending more time at home and engaging in indoor activities. Accordingly, children's self-care and social skill development was influenced positively. The pandemic's impact on children's social skills and self-care development has not been extensively studied. However, our research revealed that engaging in indoor activities during the pandemic contributed to the children's advanced development in these domains. The quality time spent with family during the pandemic appeared to be a positive factor influencing children's developmental outcomes.

In terms of the child's age of walking, a reverse relationship was observed with their social skills and self-care developmental levels during the pandemic. Those who started walking at a younger age demonstrated more advanced skills (25.5% for those who started walking before 12 months, 12.5% for those who started walking after 15 months). Prior research emphasized that children make rapid progress in various developmental domains, including social skills, during the period when they start walking (12–24 months)^43^. Additionally, children who begin walking earlier tend to exhibit better motor skills and skeletal development, allowing for more independent movement and exploration, contributing positively to their overall development.

Notably, children who were not assisted by fathers in terms of eating demonstrated more advanced social skills and self-care development. This self-sufficiency in eating is essential for self-care development. Children with strong self-care skills gain confidence in their abilities, which contributes to their social development. This suggests that during the pandemic, children who were allowed to feed themselves experienced more advanced development. Literature has consistently shown that children with strong connections to their fathers tend to exhibit higher overall development^44,45^.

The COVID-19 pandemic prompted increased engagement in indoor activities and family time, which in turn led to more advanced self-care and social skill development in children. As children spent more time at home due to restrictions and social isolation, their involvement in home activities increased. A qualitative study by Demir Öztürk et al. found that children engaged in indoor games and spent time with siblings during the pandemic, followed by activities with their mothers^46^. This emphasis on family time contributed to children's developmental outcomes.

In conclusion, our study highlighted various factors influencing children's social skills and self-care development during the COVID-19 pandemic. Factors such as gender, age of walking, sibling presence, and paternal involvement were found to contribute to the varying levels of development observed in these domains. The findings underscore the importance of understanding how these factors interact and influence children's overall development, offering insights for educators, caregivers, and policymakers.

## Conclusion and recommendations

The advanced level of overall child development is positively influenced by factors such as the child's younger age, increased family income, and paternal involvement in the child's play activities. Younger age of the child positively impacts both the overall developmental level and other sub-dimensions. During the pandemic, older children staying at home were more negatively affected in their development. In situations like the pandemic, where children are forced into home isolation, families arranging more activities with their children can contribute to their development.

### Limitations of the research

Conducting the research only in a city center constitutes the limitation of the research. Cross-sectional studies are not like follow-up studies and the results have limitations.

## Data Availability

This study was prepared by using the data of the master’s thesis named “Examination of psychomotor development of children who are 6–36 months in the covid-19 stay at home period”. The datasets generated and/or analyzed during the current study are not publicly available due to limitations of ethical approval involving the participants’ data and anonymity but are available from the corresponding author on reasonable request.
